# Health professionals' and researchers' perspectives on prenatal whole genome and exome sequencing: 'We can't shut the door now, the genie's out, we need to refine it'

**DOI:** 10.1371/journal.pone.0204158

**Published:** 2018-09-21

**Authors:** Ruth Horn, Michael Parker

**Affiliations:** Department of Population Health, The Ethox Centre and Wellcome Trust Centre for Ethics and Humanities, University of Oxford, Headington, Oxford, United Kingdom; Stellenbosch University Faculty of Medicine and Health Sciences, SOUTH AFRICA

## Abstract

The Prenatal Assessment of Genome and Exomes (PAGE) project is a UK-wide study aiming to gain a better understanding of genetic variants causing developmental problems during pregnancy. A further aim of the study is to provide an evidence-base for the introduction of prenatal whole genome and exome sequencing (PWGES) into prenatal diagnostics provided by the NHS, which is expected in 2018. This paper presents the findings of a qualitative interview study undertaken with 20 health professionals and researchers involved in the PAGE project, and explores their implications for understandings of ‘good practice’ in the uses of prenatal genomics clinically. A number of critical issues are identified that will need to be addressed in the development of a model of good ethical practice for prenatal genomics: consent, management of expectations, return of results, and professional duties in the context of PWGES. The analysis presented identifies and illustrates a great deal of complexity and qualitative richness in these issues as they arise in the day-to-day work of genomics professionals. Inclusive, critical discussion of these findings, together with the findings from other empirical studies, normative analysis and scientific discoveries resulting from PAGE, will be required to inform the development of appropriate guidelines of good ethical practice that address the needs and concerns to be encountered in daily clinical practice.

## Introduction

The introduction of genomic medicine into prenatal care and its potential to provide information about the developing foetus is leading to a radical transformation of the practice of reproductive medicine. Existing prenatal testing technologies–karyotyping, microarrays, single gene and panel testing—enable the detection of certain chromosomal abnormalities (e.g. Down’s Syndrome, Turner Syndrome) or single gene disorders (e.g. cystic fibrosis, muscular dystrophies). Such technologies target a limited range of defined abnormalities. Recent advances in prenatal whole genome and exome sequencing (PWGES), however, with their greater resolution have the potential to detect a much wider range of genomic abnormalities [1–4]. Moreover, the insights such approaches provide into the genomics of the foetus and its development also offer the potential for a better understanding of the nature and causes of structural developmental abnormalities and can, hopefully, inform the development of effective interventions.

The Prenatal Assessment of Genome and Exomes (PAGE) project is a UK-wide study (of 1000 families in which a structural abnormality has been identified in pregnancy but no diagnosis has been possible) aiming to gain a better understanding of genetic variants causing developmental problems during pregnancy [5, 6]. Analysing in detail every single gene in the parents’ and foetus’ DNA, the aim of PAGE is both to provide diagnoses for the recruited families and to improve prenatal diagnostics, allowing better genetics-derived prognoses and more informed reproductive decision-making [7]. A further aim of the study is to provide an evidence-base for the introduction of PWGES into prenatal diagnostics provided by the NHS, which is expected in 2018.

In addition to the scientific and clinical challenges of achieving this, it was recognised at an early stage by the PAGE team that the introduction of new reproductive genomic technologies in the context of the PAGE study, and also subsequently into clinical care, was likely to present a number of practical ethical problems. These problems would need to be identified, understood and carefully analysed in order to advance the development of models of good ethical practice. In recognition of this, the PAGE study included embedded ethics support and advice from the outset and also incorporated an ethics and social sciences research programme combining a critical review of the existing literature on ethical issues in prenatal genomics [8], a series of workshops with health professionals and researchers involved in PAGE and a qualitative interview study exploring professionals’ views, experiences and concerns with regard to PWGES.

In this paper, we report the results of the interview study undertaken with 20 health professionals and researchers involved in the PAGE project. The interviews explore the professionals’ views and experiences, and the ethical implications of these experiences for whole genome and exome sequencing during pregnancy which is expected to be rolled out in clinic in 2018. More specifically, professionals’ views were obtained on consent, management of expectations, return of results, and professional duties in the context of PWGES. In this paper we present themes that emerged in our analysis of the interviews. We conclude with a discussion of the findings in the light of existing literature and reflect on ways forward for the development of good ethical practice when WGES will be offered as part of prenatal care in the clinic.

## Methods

In 2016 and 2017, 20 semi-structured interviews were conducted with health professionals and researchers involved in the PAGE project. The interviews represent; a) ‘clinical’ perspectives (n = 12) from those who recruited couples for the PAGE study and who are in contact with women and couples in the clinical setting (5 midwifes, MW; 4 genetic consultants, GC; 2 patient representatives, PR; 1 research assistant involved in patient recruitment, RA); and b) ‘research’ perspectives (n = 8) from those involved in the development of the sequencing technology or the interpretation of the genomic data gathered (2 laboratory staff, LS; 6 genetic scientists, GS).

The study was approved by the Central University Research Ethics Committee at the University of Oxford (R44366/RE001) and the Health Research Authority (IRAS project ID:209003). Participants were recruited through the PAGE email list, which is a comprehensive list of all health professionals and researchers (n = 90) involved in the project. Each participant received an information sheet about the study and written consent was taken prior to the interviews.

Interviews were conducted by RH who is an experienced qualitative researcher. Each interview lasted approximately 45 minutes, was audio recorded and transcribed verbatim. The aim of the interview study was to better understand professionals’ experiences with the PAGE project and more broadly their views on PWGES. For this purpose, the interviews explored professionals’ experiences with patient recruitment and informed consent, management of parents’ expectations, return of results, and professionals’ views on their duties in the context of PWGES. The topic guide was conceptualised by both authors. The choice of interview topics was guided by our initial literature review and two workshops, which were organised and facilitated by both authors as part of the ethics and social sciences research programme of PAGE. Each workshop involved 30 professionals from PAGE and provided opportunities for the exploration of practical ethical issues arising from both the literature and participants’ experiences of using prenatal genomics.

The theoretical thematic analysis was driven by our interview questions and involved numerous readings of the transcribed interviews. To begin, RH coded each segment of data that was relevant to or captured interesting information about the questions in our interview guide. The initial codes together with representative interview fragments were then reviewed and recoded, and comparable recurrent themes and patterns in the data were identified and refined. The themes that RH identified with regard to the interview topics were discussed on a regular basis with MP as the analysis progressed. Both authors interpreted the coded data. Participants were 80 professionals from PAGE, including some of the interview participants, and other national stakeholders in the field of prenatal genomics. The conference provided an opportunity for PAGE professionals to comment on the study and whether our findings and interpretations adequately reflect their attitudes and experiences with the PAGE project, this provided external feedback concerning the accuracy of the analysis.

## Results

### The complexity of achieving valid consent

The importance of consent is emphasised in both clinical and research guidelines [9–11]. Genomic research in the prenatal context, however, presents important challenges for the achievement of valid consent because of the complexity of genomics and the intensity of emotional stress for parents when foetal abnormality is detected or suspected during pregnancy [8]. Our interviews showed that timing of the discussion and the way complex information is transmitted are crucial to obtain valid consent.

#### The timing for seeking consent

The PAGE project, adopted a trio design. That is, the study involved a comparison of the exomes of both parents and the foetus [7]. The foetal sample for PAGE was obtained from a sample taken during routine amniocentesis: an invasive procedure offered as part of the clinical screening programme aimed at better understanding abnormalities observed on ultrasound. Participants for PAGE were recruited at several centres. In some, participants were recruited before amniocentesis, in others recruitment to the PAGE project took place after amniocentesis had been completed.

Health professionals who recruited patients prior to amniocentesis first introduced the PAGE project to them at the time the consultant offered the amniocentesis to the parents. Formal consent was obtained a few days later when the parents came back for the amniocentesis procedure. Health professionals we interviewed, and whose centres had adopted this approach, thought that the time given to parents between the initial contact and the actual recruitment was very important:

*People need time to think about things*, *they want a day or two to go away and consider whether they want to go ahead with the amniocentesis*, *and so in those situations we would talk to them also a little bit about PAGE*, *give them the information to take away with them and then when they come back [we would take consent]*. (MW1)

One genetic consultant who recruited parents prior to amniocentesis expressed concerns about post-amniocentesis recruitment (the approach taken by some other centres):

*Once they [women]’ve had an amnio they’re in a bit of pain and […] it’s almost like*, *‘oh thank God that test is done*. *I think they’d be more inclined to say*, *‘Yeah do whatever you want*.*’* (GC1)

However, other interviewees who were actually involved in recruiting parents *after* amniocentesis emphasised the benefits of this approach. They explained that it is very convenient to recruit parents during the period following amniocentesis, while women are waiting to leave the hospital after the intervention. They emphasised the benefit of making use of the parents’ waiting time, and reported that these situations facilitated recruitment.

Having said this, despite their tendency to emphasise the benefits of post-amniocentesis recruitment, some of the interviewees acknowledged the emotional distress of parents in this particular situation:

*They are very vulnerable at that point [after amniocentesis]*. *The feedback that I’ve had from it has been that it’s been very positive for them to have someone like me spending an extra 20*, *30 minutes talking through this other process*. (MW2)

Although parents who were recruited experienced the additional time professionals spent with them as positive and reassuring, this is not the purpose of valid consent. The professionals’ acknowledgment of the parents’ vulnerability inevitably leads to questions about the extent to which it might be more difficult to achieve valid consent in the context of post-amniocentesis recruitment. It also highlights the importance of paying particular attention to the need for sensitivity to the emotional context [12].

#### Transmitting and comprehending complex information

Apart from these important contextual features of prenatal genomics, health professionals also highlighted the challenges of achieving valid consent to genomic testing *per se* and acknowledged its importance as an issue.

Several midwives involved in recruitment for PAGE, questioned the ability of parents to really comprehend the study information at a time when they are anxious about their pregnancy:

*We’re seeing families at an extremely vulnerable time*. (MW2)

*It’s quite a stressful time and quite an upsetting time so it can be difficult to sort of get a sense of*, *you know*, *how much they really understand*. (MW3)

Another midwife confirmed:

*If you’ve just come here and there’s a suspected diagnosis*, *it’s been confirmed*, *and then you’ve had that discussion about what it could mean for your baby […] I don’t know how much you take in*. (MW1)

This reflects the findings of other studies which have highlighted the difficulty of communicating complex genomic information to a parent when they are under stress after a foetal abnormality has been detected [13, 14].

Importantly, health professionals distinguished between parents’ understanding of the procedural aspects of participation and their understanding of the broader implications of prenatal genomics. They were confident that parents who consented to participate in PAGE understood the procedure, but had doubts about parents’ understanding of the meaning and scope of genomic research and medicine.

*I think they [parents] fully understand the process that they have been enrolled into*. *I’m very happy that they understand what they’ve signed up to as far as the process is concerned […]*. *But genetics is a complex topic*. *So how much they properly understand when I’m talking about finding specific genes*, *um*. (MW2)

An interview study explicitly exploring the experiences of parents recruited for PAGE, confirmed the difficulties they faced in fully comprehending the meaning of genomics [13]. Reflecting discussions in the academic literature [8] about the best ways to *assess how much [parents] do fully understand* (MW4), our interviewees argued that consent should be seen as an ongoing process and participants should be provided with as much information as they require at several stages along the journey from initial identification of the anomaly through to genomic testing [15–17]. As one of the midwives stated:

*I think it’s repetition as well*. *You know*, *if they’re able to talk to a few people about it and ask questions*, *and have an ongoing opportunity to ask questions*, *they always have my contact number and things so I think that kind of can help with continuing to develop their understanding*. (MW3)

It was recognised however that continuity is sometimes difficult to achieve. Taking PAGE as an example, as research staff changed throughout the project, continuity in the participant contact, and hence in the consent process, could not always be guaranteed.

### Management of expectations

#### The hope to ‘get an answer as to what went wrong’

In addition to the complexities of genomics and the stressful context in which consent is obtained, the difficulty of communicating effectively about the implications of participation in the PAGE study was also compounded by parents’ strong clinical reasons for choosing to participate in the study. According to the health professionals interviewed, parents consented to the study because they wanted to understand the abnormality observed in their foetus and its implications for future pregnancies. Yet, it was clear from the outset that in many cases these expectations would not be possible to meet because; a) the interpretation of sequencing data to explain an abnormality seen on a scan is difficult and cannot always be guaranteed [7], and b) as discussed above, it is difficult to comprehensively communicate complex genomic information to parents. In order to avoid disappointment and misunderstanding, health professionals involved in recruitment considered it to be crucial to discuss parents’ expectations and to ensure that the limits of genomics were understood prior to taking consent [18, 19]. Our interviewees acknowledged the difficulties of achieving this, however. They consistently reported that parents wished to get *‘an answer as to what’s gone wrong with baby and why*. *[They] just want information’* (RA).

In the PAGE project, the challenges of interpretation and of establishing a reliable and rapid pipeline for testing and feedback in the context of the lifetime of the project were recognised. Hence, a decision was made that results would not be returned to women and their partners until after the pregnancy. This was explained, along with the reasons for it, at the time of consent. The health professionals reported that this was not always well received by some couples:

*‘Some have raised their eyebrows and said*, *‘Well what’s the point*? *If you can’t give me any information*, *then I’m not sure we want to do this’* (MW4).

In general, however, interviewees reported that the majority of parents accepted this and were mainly hoping to be given *‘information which could be relevant for future pregnancies or for that child*, *if the child survives’* (MW3). The health professionals described that parents *‘want a good thing to come out of a bad situation’* (MW4) and *‘be able to contribute in some way to the development of knowledge’* (MW3). The desire for more information which the professionals we interviewed reported as the main drive for parents’ participation in PAGE, was confirmed also by a study exploring the experiences of parents who took part in the PAGE project [20].

#### Preparing for genomic findings and no findings

Health professionals reported that even for those participants who acknowledge that feedback would only be relevant to future pregnancies, the wish to get *‘to the bottom of what’s actually caused [the abnormality]’* (MW2), sometimes led to high expectations that would be difficult or impossible to meet in genomic research and medicine. This mirrors the findings of Cacioppo et al. who also observed frequent discrepancy between parental expectations and actual results in genomic research studies [18]. The complexity of genomic information, and the challenges of establishing the meaning and the interpretation of research results mean that, as one genetic consultant noticed, parents who hope (as most of them will) to get an answer as to what is wrong with their baby need to have their expectations managed prior to receiving results:

*Sit down and talk them through it*, *because probably the most important thing is to have the conversation before [you get] the results*. (GC2)

Another interviewee, a genetic scientist, supported this, arguing that misunderstandings and false expectations can often be avoided through thorough counselling:

*The problems arise when people haven’t been told in advance*, *so then they look*, *‘where did that come from’*? *[…] I think it’s important that before the test is undertaken the counselling explores some options with some examples potentially for them*, *because that makes it real for people*. (GS1)

One of the consultants interviewed emphasised the importance of discussing all possible results with the parents in advance and of helping them think through possible decisions, or ‘exit strategies’, before feeding back results:

*I detail the three possibilities [no finding*, *uncertain finding*, *finding] that might come*, *and I go through each of those individual possibilities with the couple and make sure they almost have considered an exit strategy for each of those*. (GC1)

Notwithstanding these difficulties, health professionals who recruited parents were confident that, by the time consent was given, they understood the likelihood of receiving no further information regarding the foetal abnormality identified,

*[…] because [the difficulty to get clear results] is explained well to patients initially and we’re kind of managing their expectations*. (MW4)

*Within medicine there is always an element of uncertainty [*…*]*. *And I think most families know that this testing might not give them an answer*. (MW3)

The health professionals’ confidence with regard to their management of parental expectations can, to a certain extent, be explained by the set-up of the PAGE study, which required that it was explained to parents at the outset that study results were to be fed back to them only after pregnancy. Furthermore, PAGE opted to return only information on variants that explained the structural abnormality seen on the ultrasound scan; that is, no incidental or uncertain findings were communicated to parents [7].

### Return of results

The interpretation of genomic variants in the prenatal context is difficult because the phenotype may not be fully detectable on screen or the penetrance of the variant is unknown [21]. Sequencing the whole genome and exome of a foetus will generate a vast amount of variants of which the significance is unknown or uncertain. Only few variants will be of known significance. The still limited knowledge in the new field of prenatal genomics means that it is difficult to target the sequencing, which implies that incidental findings will be generated [13].

#### The right to know or not to know

The health professionals and researchers in the PAGE project have had early experience with uncertain or incidental findings and reflected on their wider implications for clinical practice. In the interviews, they placed important value on the parents’ right to choose which information they wish to receive.

*It’s [about] whether they have a choice about the information they want to know*, *which they should have really*. (MW1)

It was their view that even though in the particular research context of the PAGE study no incidental or uncertain findings were being reported, once prenatal genomics is introduced into clinical practice, it will not be right to hold back information:

‘*If the information is out there*, *people are entitled to know it*.*’* (MW2)

Yet, they also acknowledged that:

*[…] blissful ignorance sometimes is not a bad thing*! *[Only] if we do the test we’re going to have to report it*. (GC2)

The key concern for the professionals we interviewed was patient centred-ness. For example, as much as they emphasised the importance to give parents the choice to decide what they want to know, they also considered it important to also give them a choice not to know:

*I don’t think we should force genetic information on people that don’t want it […] an opt-in and opt-out system would be good for that sort of thing*. (LS1)

#### The ‘usefulness’ of information

Despite the fact that the health professionals and researchers placed a great deal of emphasis on patient choice, most of them thought that the scope of the right to know should be limited by the ‘usefulness’ of information for the decision-making. ‘Usefulness’ here was mostly defined in terms of ‘clinical usefulness’.

*I think [actionable findings] should definitely be fed back*. *[But if] the penetrance of a certain mutation isn’t 100% […]*, *just because someone tells me I have a mutation doesn’t mean that I’m going to die from that mutation*.*’ I think not feeding back [those] findings is largely the right thing to do*. (LS2)

One of the genetic scientists took an even stronger stance when arguing that feedback should not be guided by the principle of autonomy if there is no clinical usefulness of the result:

*I think it’s a wider context*, *and the autonomy is a positive side effect but I wouldn’t put it as a priority*. *What is the clinical value of a Class 3 [variant where pathogenicity or lack of it cannot be assigned based on current knowledge]*? *Not much […] there’s nothing we can do about it in the present time*. (GS1)

Interestingly, some of our interviewees were willing to go further than limiting information to that which was ‘clinically useful’ to that which was ‘useful’ in the specific context at hand. That is, to the actual situation, the ‘here and now’. This was particularly true of health professionals who had face-to-face contact with parents. They tended to emphasise the importance of only returning data relevant to the actual pregnancy or early childhood:

*There is sometimes too much information*, *it’s not useful for the situation that they’re in*. *[…] They either want to be prepared for when their baby is born so that they know the challenges their baby might face*, *or it’s because they’re considering the option of termination*. (GC3)

#### The broader impact of PWGES

In cases where the available genomic information would not inform pregnancy or childhood decisions, but might be relevant to the later life of the child, one of the midwives was more hesitant:

*I think it’s quite easy if it’s a physical thing and it’s there from birth*, *but something that might develop*, *that’s really opening up a whole different Pandora’s box*, *isn’t it really*. (MW5)

More generally, where there was uncertainty about the future or actual significance and meaning of the results, our interviewees argued that it would be better not to share this information. According to some of the laboratory staff, *‘when we sequence there’s a fairly high error rate to the technology we’re using’* and *‘there are a lot of technical hurdles that need to be overcome for [results to be fed back during pregnancy]’* (LS1). Health professionals and researchers were concerned that the uncertainty *‘could create more problems than we’re trying to solve’* (MW2) and *‘create unnecessary anxiety’* (MW4). Unless the broad public will be *‘educated’* and *‘understand [genomics] at a basic level through media or other influences’* (RA), they considered it difficult to give parents every information, particularly where this was deemed it to be of no clinical utility.

Taken together, this shows that health professionals and researchers believe that patients choices about information and feedback should determine what information is returned and when, but that the scope of their autonomy ought to be limited to information with clinical relevance. Our interviewees acknowledged the challenges of deciding on feedback about uncertain information within the context of even this more limited scope, however.

#### Turnaround times

Within the context of the PAGE project itself, study results were fed back only after pregnancy. Health professionals expressed some concern about the fact that feedback within the research context was taking around a year. This was particularly difficult, they argued, for parents who had miscarried:

*We’ve had some patients who are sort of OK not to find out during the pregnancy but they don’t want to find out a year later because […] they will try to move on and forget about what happened*. (GC4)

A study exploring parents’ experiences within PAGE [20] confirmed that the long turnaround time in the study was difficult for some of the parents recruited.

Another difficulty faced by health professionals in PAGE arose in those situations in which they had to contact parents who–despite the earlier anomaly at ultrasound–had had a seemingly healthy baby, but where the WGES has shown an association between a genetic variant and the initially detected structural abnormality:

*You can’t really just ring them up and say*, *‘Oh by the way*, *is your child normal now*?*’ […] do you just poke all of that [worry] up with a stick when you come back with a result*? *Are they grateful for it*? (GC1)

When prenatal genomics is introduced into clinical practice more widely–from later in 2018, results will be fed back during pregnancy in order to inform parental decision-making and problems related to a long turnaround time will not arise in the same way.

The attitudes and experiences of the health professionals and researchers reported in this paper mirror and confirm, at least in part, published accounts of patients’ views on the return of genomic results. Further analysis of these findings in the light of normative arguments will make important contributions to the development of ethical frameworks of good practice and help answering the question as to what findings ‘ought’ to be fed back.

### Professional duties in the context of PWGES

#### The duty to improve knowledge and facilitate informed decisions

Despite the difficulties discussed above, overall, health professionals and researchers valued PAGE, and more broadly prenatal genomics. The biggest benefit associated with PWGES was the possibility it offered to provide answers to parents that would not otherwise be available.

*Often our women will have an invasive testing*, *have a karyotype or array and actually don’t get any answers or explanations*, *and I think PAGE can add an additional layer to that and give us a bit more information*. *[…] There is a thirst for knowledge and a thirst for explanation of why some of these babies do develop problems in pregnancy*. (MW4)

Our interviewees emphasised the importance of PWGES as a way of helping parents to prepare for difficult situations and make informed decisions:

*[It] gives them a much better idea of how it will affect their children […] then preparing and coping with it might be better*. (GC4)

*[It offers] more information to make a more informed choice*. (PR1)

They perceived the provision of information not only as one of the benefits of PAGE, but also as one of their duties towards parents:

*I have an obligation to make sure that the parents have all the information they need to make the best decision for their child and their wider family if they’ve got one*. (GC3)

Another important aspect associated with PAGE, was the opportunity to ‘*find out more about how we can avoid major abnormalities’* and *‘eradicate [serious disability] not something like Down’s syndrome*, *but where I see sort of quadriplegic children*, *that’s not the same thing*.*’* (MW2). That is, to help future parents and children as well as those currently being seen. Generally, the interviewees, particularly the researchers, saw knowledge generation and advancement of technology as their duty, and as the major advantage of PAGE:

*PAGE will progress the technology or help to establish the new technology of using next generation sequencing for this kind of diagnosis*. (GS2)

Both clinical and scientific staff considered it as their obligation to contribute to the growth of knowledge and the development and improvement of technology:

*I think as a scientist I’m in favour of progress and change*, *and I want to work on things that are going to improve the world and the way healthcare is operated*. (LS1)

*I think if we have the potential to find out information […] we can’t really shut the door now*, *the genie’s out*, *we need to refine it*. (MW4)

#### The duty to protect patients from technology

Despite their enthusiasm and their sense that research and innovation are important parts of their work, the health professionals and researchers we interviewed were realistic and recognised the importance of paying close attention to their social and ethical implications. Amidst the enthusiasm about innovation, some professionals mentioned that the limits and scope of the new technologies require to be discussed by society:

*We have to progress*, *we can’t hold ourselves back unnecessarily*, *and we need to make a decision as scientists*, *as ethicists and as a society as to what we think is an acceptable use of this technology*. *But I absolutely think that it’s right to push the boundaries and to do the best job that we can for these people*. (LS1)

Also others expressed concerns about the fast evolving field of genomics:

It is opening a sort of new chapter in medicine and we’re not fully understanding the implications. […] I think there is the potential for stress and anxiety if we don’t quite understand what we’re dealing with […] are we opening a can of worms?

(MW4)

The risk of *‘opening a can of worms’* as a metaphor for the vast amount of data that cannot be fully understood and the yet unknown implications of prenatal genomics, was mentioned by several interviewees. One of them also referred to the *‘potentially toxic knowledge’* (GC1) WGES may generate. Such knowledge was discussed as particularly problematic in the case where WGES would be offered by private providers without comprehensive counselling:

*I think what’s worrying me is that [in the private sector] it’s not under a controlled environment*. *[…] The risk that is would become too easily accessible […] not for the right reasons and not properly explained*. (GC2)

In order to prevent the loss of control over new technologies, one of the interviewees, a genetic consultant, reminded of their obligation to continuously question the aim of scientific progress:

*[It] is our job to protect the patients from scientists*, *almost*. *Because if you ask a scientist […] their eyes will light up and they will just go deep down a rabbit-hole*, *‘Oh wow*, *let’s look even further*, *let’s dig*, *let’s lift up the mattress and properly look underneath*! (GC1)

#### The duty to provide education and resources

Other interviewees also mentioned the role education plays in how technology is used and understood. They acknowledged their obligation to contribute to this education:

*We can influence the course of action by providing open*, *clear information*, *and not hiding evidence to others; just facts and data and then people can make up their minds*. *[…] I think our responsibility is also to train the clinical teams*, *the frontline people*, *to make sure they have the facts right and they understand what they are offering the patients or not*. *[…] I think mostly of our responsibility is how we educate people about using this information*. (GS1)

However, many of the interviewees were concerned about how to guarantee education in view of the complexity of the fast evolving field of prenatal genomics and increasingly limited resources in the National Healthcare Service (NHS):

*The huge challenges for us introducing this technology*, *is education […] there would have to be almost a mass*, *you know*, *a mass discussion about prenatal ethics*, *about education*. *[…] if you are delivering this test then you have a responsibility*. (GC1)

*We don’t have enough genetic counsellors working prenatally […] we do not have the support that’s required*. *[…] You do need the resources there […] the whole genomic testing will be extremely costing*. (PR2)

Limited resources are an issue also in relation to access to genomics services. While the costs of the sequencing itself have dropped in the past years, the costs of data management including analysis, interpretation and storage are growing [8]. In the interviews, the genetic scientists expressed some concern about equal quality standard across the country, whereas clinical staff expressed worries regarding data protection:

*It does slightly worry me on the other hand that we’ve got all this information and we’re storing it*, *and it’s anonymised but it’s going out of the country […] it’s meant to be secure but I’m not sure anything is secure now*. (MW5)

Our interviewees emphasised research companies’ and public authorities’ obligations to meet the costs for data management, as they were worried that otherwise the quality of the information obtained as well as data protection cannot be guaranteed [22].

## Discussion and ways forward

As the PAGE study comes to an end, it is expected that PWGES will increasingly be available through the NHS from 2018. Our interviews have highlighted important questions and practical ethical issues that will need to be taken into account in the development, implementation and evaluation of models of good ethical practice in prenatal genomics. These aspects overlap to some degree with those identified in the preceding literature review on PWGES [8] but are given a much greater degree of nuance and depth by the analysis presented above. From a bird’s eye view, the issues at stake are: consent, management of expectations, return of results and professional duties in the context of PWGES. Our findings illustrate a great deal of complexity and contextual variation across these issues. In the following we reflect on these issues in the light of our interviews as well as existing literature, and consider what might be required to establish models of good ethical practice in the use of prenatal genomics clinically.

The analysis presented here highlights the fact that parents’ consent to undergo PWGES is strongly influenced by the timing of and context within which information about this test is provided. It is also apparent that parents’ understanding of complex genomic information can be framed by unrealistic expectations and the emotionally distressing situation parents find themselves in when foetal abnormality has been detected. As our interviewees confirmed, it is clear that any effective approach to consent is going to need to be interactive and on-going, and that time needs to be given to parents to consent. Only then will it be possible, to provide information that is tailored to the parents’ needs and receptivity at an emotionally stressful moment and as part of an on-going conversation [23]. The required model of consent will need to be a ‘communicative process that is consent‐in‐action’ and an ‘opportunity to discuss the return of results in advance’ [24]. For this purpose, and recognising the time-constraints of pregnancy, continuity in the care and support provided to parents need to be guaranteed. Guidelines on the implementation of PWEGS will be required to help health professionals, but also researchers, to make structured and well-considered judgements about ‘when’ the test should be offered, ‘how’ complex information is communicated and ‘what’ parents actually understand.

Similarly, a one-size fit all approach regarding the return of results is unlikely to be appropriate in the light of different patient experiences and preferences and in the context of the variability of the definition of clinically or personally ‘useful’ data. Studies have shown that patients wish to have a choice in decisions about what information is fed back [21, 22]. There are some interesting tensions, however. For example, while some patients might wish to receive only ‘clinically’ useful information, other patients might wish to receive every information they personally judge useful; in some cases, this may include variants of uncertain significance or even their whole sequence. These empirical findings challenge a one-size-fit-all approach, and call for involvement of the parents in pre‐test counselling discussions [23]. However, some limits will clearly need to be placed on what information should be returned in order to both be manageable in the clinical setting and also to avoid harm to parents or the future child [25]. There are a number of important and, as yet, unresolved questions about how feedback ought to be managed. To what extent should feedback focus solely on information relating to the identified anomaly on ultrasound? To what extent should broader findings be returned? How should such decisions be made and by whom? It is likely that feedback will only take place where there is ‘clinical utility [26, 27]. In the prenatal context, however, it is often difficult, with the exception of few conditions, to predict or foresee whether and if so, how, an identified variant will affect the child later in life. It is clear that these are not solely scientific or clinical questions but are strongly value-laden [8]. It might be that, for example, an agreed list of genomic variants to be reported could be produced, similar (in approach rather than in specific content) to the recommendations of the American College of Medical Genetics and Genomics [27]. However, as Green et al. acknowledge, it may be “difficult to reach consensus on a specific list of variants that meet a threshold for disclosure” [27] and appropriate patient-centred variation may also be an important consideration. Whichever approach is adopted, possible findings as well as limitations of what information will be reported should be discussed during counselling and prior to taking consent [28]. Key to this is going to be the management of expectations to reduce conflict and uncertainty in the relations between clinicians and parents [18].

It may be that confidence in clinical practice or research in prenatal genomics may best be built by providing clear knowledge and improving technology. Our interviewees felt a strong obligation to advance genomics medicine through research participation in order to facilitate the development of better diagnosis, and ultimately also treatment for genetic conditions. Yet, they were aware of the risks of over-emphasising the benefits of genomics as well as driving scientific advances without carefully assessing the broader individual and social implications [29]. The health professionals and researchers of PAGE felt that patients need to be protected from ‘toxic’ knowledge [30] and technology. One way of doing this is by providing broader genomics education. Given the interviewees’ comments above about the important role of counselling, it is clear that sufficient resources need to be made available not only for high quality technology and data curation but also for effective and well-resourced counselling in the prenatal context. This includes the provision of sufficient numbers of staff, available time for continuous information, and education of staff as well as the broad public [31]. Finally, putting in place multi-disciplinary national review panels validating the variants identified in order to guarantee coherent results across the country has proven very useful and consensus-building in the PAGE study. Such panels have also been adopted in other studies such as the UK’s 100,000 Genomes Project allowing for equal quality standard of data interpretation across the various research sites [32].

## Conclusion

The interviews with health professionals and researchers involved in the PAGE project reported here have highlighted a number of critical issues that need to be addressed in the development of a model of good ethical practice and implementation of prenatal genomics services in the UK National Health Service: consent, management of expectations, return of results and professional duties. Our findings have highlighted some of the complexity and qualitative richness of these issues as they arise in the day-to-day work of genomics professionals. As prenatal genomics moves into clinical practice, guidelines need to be developed to address the following questions: What is required for effective, valid consent for prenatal genomic testing? When and how should consent be sought? What and how much information should be fed back to parents? What data should be stored, how, and with whom should it be shared? What can be done to guarantee equal quality standard of data interpretation and validation across the country? What are the obligations of health professionals, researchers and other stakeholders to patient/participants (and their families)? What and how many resources, in terms of staff, time and education, are needed to secure high quality counselling, and how can this be achieved? In order to address these questions, the empirical findings presented in this paper as well as those by other researchers in the field need to be taken into account and further discussed in the light of normative arguments. In a next step, various stakeholders in the field, clinical staff, scientists, and patients, in collaboration with ethicists and sociologists should be involved in the development of appropriate guidelines of good ethical practice that address the needs and concerns to be encountered in daily clinical practice.

## References

[1] Vora NL, Powell B, Brandt A, Strande N, Hardisty E, Gilmore K, et al Prenatal Exome Sequencing in Anomalous Fetuses: New Opportunities and Challenges. Genetics in medicine: official journal of the American College of Medical Genetics. 2017;19(11):1207–16. 10.1038/gim.2017.33 PMC5675748 28518170

[2] Best S, Wou K, Vora N, Van den Veyver IB, Wapner R, Chitty LS. Promises, pitfalls and practicalities of prenatal whole exome sequencing. Prenatal diagnosis. 2017. 10.1002/pd.5102 PMC5745303 28654730

[3] Drury S, Hill M, Chitty LS. Recent developments in non-invasive prenatal diagnosis and testing. Fetal and Maternal Medicine Review. 2014;25(3–4):295–317.

[4] Kitzman JO, Snyder MW, Ventura M, Lewis AP, Qiu R, Simmons LE, et al Noninvasive whole-genome sequencing of a human fetus. Science translational medicine. 2012;4(137):137ra76–ra76. 10.1126/scitranslmed.3004323 22674554 PMC3379884

[5] Prainsack B, Reardon J, Hindmarsh R, Gottweis H, Naue U, Lunshof JE. Personal genomes: Misdirected precaution. Nature. 2008;456(7218):34–5. 10.1038/456034a 18987720

[6] Wellcome Sanger Institute. PAGE, Prenatal Assessment of Genomes and Exomes [Available from: https://www.pageuk.org/.

[7] Hillman SC, Willams D, Carss KJ, McMullan DJ, Hurles ME, Kilby MD. Prenatal exome sequencing for fetuses with structural abnormalities: the next step. Ultrasound in Obstetrics & Gynecology. 2015;45(1):4–9. 25157891 10.1002/uog.14653

[8] Horn R, Parker M. Opening Pandora's box?: ethical issues in prenatal whole genome and exome sequencing. Prenatal diagnosis. 2018;38(1):20–5. 10.1002/pd.5114 28695688 PMC5836985

[9] World Medical Association. Declaration of Helsinki (Human Experimentation: Code of Ethics). 1964.

[10] General Medical Council. Good practice in research and consent to research. 2013.

[11] General Medical Council. Good medical practice. 2013.

[12] Helmreich RJ, Hundley V, Norman A, Ighedosa J, Chow E. Research in Pregnant Women: The Challenges of Informed Consent. Nursing for Women's Health. 2007;11(6):576–85. 10.1111/j.1751-486X.2007.00250.x 18088295

[13] Quinlan‐Jones E, Kilby MD, Greenfield S, Parker M, McMullan D, Hurles ME, et al Prenatal whole exome sequencing: the views of clinicians, scientists, genetic counsellors and patient representatives. Prenatal diagnosis. 2016;36(10):935–41. 10.1002/pd.4916 27550507

[14] Vetro A, Bouman K, Hastings R, McMullan DJ, Vermeesch JR, Miller K, et al The introduction of arrays in prenatal diagnosis: A special challenge. Human Mutation. 2012;33(6):923–9. 10.1002/humu.22050 22508381

[15] Yu JH, Jamal SM, Tabor HK, Bamshad MJ. Self-guided management of exome and whole-genome sequencing results: changing the results return model. Genetics in medicine: official journal of the American College of Medical Genetics. 2013;15(9):684–90. 10.1038/gim.2013.35 PMC4010112 23619276

[16] Allmark P, Mason S. Improving the quality of consent to randomised controlled trials by using continuous consent and clinician training in the consent process. Journal of Medical Ethics. 2006;32(8):439–43. 10.1136/jme.2005.013722 16877621 PMC2563382

[17] Gupta UC. Informed consent in clinical research: Revisiting few concepts and areas. Perspectives in Clinical Research. 2013;4(1):26–32. 10.4103/2229-3485.106373 23533976 PMC3601699

[18] Cacioppo CN, Chandler AE, Towne MC, Beggs AH, Holm IA. Expectation versus Reality: The Impact of Utility on Emotional Outcomes after Returning Individualized Genetic Research Results in Pediatric Rare Disease Research, a Qualitative Interview Study. PLoS ONE. 2016;11(4):e0153597 10.1371/journal.pone.0153597 27082877 PMC4833284

[19] Klitzman RL. Misunderstandings Concerning Genetics Among Patients Confronting Genetic Disease. Journal of genetic counseling. 2010;19(5):430–46. 10.1007/s10897-010-9307-z 20512408 PMC2945403

[20] Quinlan‐Jones E, Hillman SC, Kilby MD, Greenfield SM. Parental experiences of prenatal whole exome sequencing (WES) in cases of ultrasound diagnosed fetal structural anomaly. Prenatal diagnosis. 2017;37(12):1225–31. 10.1002/pd.5172 29049852

[21] Westerfield L, Darilek S, van den Veyver IB. Counseling Challenges with Variants of Uncertain Significance and Incidental Findings in Prenatal Genetic Screening and Diagnosis. Journal of Clinical Medicine. 2014;3(3):1018–32. 10.3390/jcm3031018 26237491 PMC4449641

[22] Muir P, Li S, Lou S, Wang D, Spakowicz DJ, Salichos L, et al The real cost of sequencing: scaling computation to keep pace with data generation. Genome Biology. 2016;17:53 10.1186/s13059-016-0917-0 27009100 PMC4806511

[23] Clarke AJ. Managing the ethical challenges of next-generation sequencing in genomic medicine. British Medical Bulletin. 2014;111(1):17–30. 10.1093/bmb/ldu017 25122627

[24] Pinxten W, Howard HC. Ethical issues raised by whole genome sequencing. Best Practice & Research Clinical Gastroenterology. 2014;28(2):269–79. 10.1016/j.bpg.2014.02.004 24810188

[25] Hall A, Hallowell N, Zimmern R. Managing incidental and pertinent findings from WGS in the 100,000 Genomes Project Cambridge: PHG Foundation 2013.

[26] Gardiner C WD, Kilby MD, Kerr B, on behalf of the Joint Committee on Genomics in Medicine Recommendations for the use of chromosome microarray in pregnancy. The Royal College of Pathologists; 2015.

[27] Green RC, Berg JS, Grody WW, Kalia SS, Korf BR, Martin CL, et al ACMG recommendations for reporting of incidental findings in clinical exome and genome sequencing. Genetics in Medicine. 2013;15(7):565 10.1038/gim.2013.73 23788249 PMC3727274

[28] Lucassen A, Parker M. Revealing false paternity: some ethical considerations. The Lancet. 2001;357(9261):1033–5. 10.1016/S0140-6736(00)04240-9 11293609

[29] Nerlich B, Dingwall R, Clarke DD. The Book of Life: How the Completion of the Human Genome Project was Revealed to the Public. Health. 2002;6(4):445–69.

[30] Bernhardt BA, Soucier D, Hanson K, Savage MS, Jackson L, Wapner RJ. Women’s experiences receiving abnormal prenatal chromosomal microarray testing results. Genetics in medicine: official journal of the American College of Medical Genetics. 2013;15(2): 10.1038/gim.2012.113 22955112 PMC3877835

[31] Dressler LG, Jones SS, Markey JM, Byerly KW, Roberts MC. Genomics Education for the Public: Perspectives of Genomic Researchers and ELSI Advisors. Genetic Testing and Molecular Biomarkers. 2014;18(3):131–40. 10.1089/gtmb.2013.0366 24495163 PMC3948600

[32] Ormondroyd E, Mackley MP, Blair E, Craft J, Knight JC, Taylor J, et al Insights from early experience of a Rare Disease Genomic Medicine Multidisciplinary Team: a qualitative study. European Journal of Human Genetics. 2017;25(6):680–6. 10.1038/ejhg.2017.37 28327571 PMC5427178

